# Visual attention, an indicator of human-animal relationships? A study of domestic horses (*Equus caballus*)

**DOI:** 10.3389/fpsyg.2014.00108

**Published:** 2014-02-13

**Authors:** C. Rochais, S. Henry, C. Sankey, F. Nassur, A. Góracka-Bruzda, M. Hausberger

**Affiliations:** ^1^Laboratoire d'Éthologie Animale et Humaine EthoS – UMR CNRS 6552, Université de Rennes 1Paimpont, France; ^2^Laboratoire d'Éthologie Animale et Humaine EthoS – UMR CNRS 6552, Université de Rennes 1Rennes, France; ^3^Polish Academy of Sciences, Institute of Genetics and Animal Breeding, JastrzebiecMagdalenka, Poland

**Keywords:** attentional processes, horses, training, reinforcement, human-animal relationships

## Abstract

As visual attention is an intrinsic part of social relationships, and because relationships are built on a succession of interactions, their establishment involves learning and attention. The emotional, rewarding or punishing, content can modulate selective attention. In horses, the use of positive/negative reinforcement during training determines short and long-term human-horse relationships. In a recent study in horses, where either food or withers' grooming were used as a reward, it appeared that only the food-rewarded horses learned the task and show better relationship with humans. In the present study, we hypothesized that this differential effect of grooming/food rewards on learning performances could be due to attentional processes. Monitoring, gazes and behaviors directed towards the trainer revealed that the use of a food reward (FR) as positive reinforcement increased horses' selective attention towards their trainer. Conversely, horses trained with grooming reward (GR) expressed more inattentive responses and did not show a decrease of “agitated” behavior. However, individual plotting of attention vs. rate of learning performances revealed a complex pattern. Thus, while all FR horses showed a “window” of attention related to faster learning performances, GR horses' pattern followed an almost normal curve where the extreme animals (i.e., highest and lowest attention) had the slowest learning performances. On the other hand, learning was influenced by attention: at the end of training, the more attentive horses had also better learning performances. This study, based on horses, contributes to the general debate on the place of attentional processes at the interface of emotion and cognition and opens new lines of thought about individual sensitivities (only individuals can tell what an appropriate reward is), attentional processes and learning.

## Introduction

Visual attention is an intrinsic part of social relationships. However, animals can deal differently with conspecifics' visual gazes depending on the gaze's characteristics *per se* (i.e., duration, frequency) or relationships between individuals (Blois-Heulin, 1999). For instance, it has long been considered that prolonged eye contact between primates is almost invariably threatening, except among closely bonded individuals (Marler, 1965). However, more recent studies have shown that eye contact is predictive of positive interactions (e.g., brown capuchin monkeys, Weigel, 1979) and mutual gazes are an integral part of a social network (Emery, 2000). In general, primates' visual attention depends on their affiliative and status relationships (Chance and Jolly, 1970). For instance, marmosets direct more gazes towards affiliative partners in a social learning task (Range and Huber, 2007). Because relationships are built on a succession of interactions associated with emotional valences (Hinde, 1979), their establishment involves learning (to associate a group member with an experience) and attention. Social affinities are thus assumed to promote selective attention and hence learning (Cousillas et al., 2006, 2008; Bertin et al., 2007).

Human-animal relationships provide a quasi-experimental framework to test hypotheses about the role of visual attention in the establishment of social relationships. Although this kind of relationship involves individuals of two different species, it is built similarly on successions of interactions whose valences induce either a positive, neutral, or negative type of relationship (e.g., Waiblinger et al., 2006; Hausberger et al., 2008). Captive and domestic animals tend to monitor actively their familiar trainer visually in both neutral and training situations (dogs: Range et al., 2009; Horn et al., 2012; primates: Anderson, 1998). Animals that have lateral eye positions, like dolphins, even turn their heads to observe their familiar trainer (Xitco et al., 2001, 2004). This sustained attention is probably involved in animals' ability to detect human cues such as attentional states (dogs: Call et al., 2003; Schwab and Huber, 2006; apes: Kaminski et al., 2004; monkeys: Hattori et al., 2007; Maille et al., 2012) or pointing gestures (for a review see Miklósi and Soproni, 2006).

Like other domestic animals, horses establish relationships with humans. In addition to the basic daily encounters that cattle or pigs may also experience (e.g., Waiblinger et al., 2006), horse-human relationships also depend on their training and working situations (Hausberger et al., 2008). Due to these interactions with humans, horses are sensitive to the attentional states of humans (Keeling et al., 2009; Proops et al., 2009; Sankey et al., 2011) and to cues given by humans (e.g., McKinley and Sambrook, 2000; Maros et al., 2008; Proops and McComb, 2010). Moreover, horses discriminate familiar from unfamiliar humans: they tend to look more often at their familiar trainer in a neutral situation (Lampe and Andre, 2012; Proops and McComb, 2012), while they spend more time monitoring (i.e., observing the human with a rotation of the head approximately 45° or more, Xitco et al., 2004) an unfamiliar human “replacing” the familiar trainer in an obedience task (Sankey et al., 2011). Although experience with humans tends to be generalized from familiar to unfamiliar humans (Hausberger and Muller, 2002; Henry et al., 2005; Fureix et al., 2009; Sankey et al., 2010a), horses still discriminate their familiar trainers (Sankey et al., 2010a; Baragli et al., 2011; Krueger et al., 2011).

The positive, neutral, or negative valence (as assumed for the horse) of the relationship influences horses' behavior (Fureix et al., 2009). Thus, training experience has been shown to be crucial: the use of positive/negative reinforcement determines short- and long-term human-horse relationships (Sankey et al., 2010a,b). Food as a reinforcement not only promotes learning and memory of the task, but also learning and memory of positive interactions with a human (Sankey et al., 2010a). Nevertheless, trainers tend to use tactile contact (McGreevy and McLean, 2011) such as patting or scratching withers as reward, in order to mimic horse-horse interactions (i.e., allogrooming). In natural conditions, allogrooming is mostly observed between preferred social partners but occurs at low rates and with very large variations of occurrences according to seasons and individuals (Waring, 2003; Feh, 2005; Mills and McDonnell, 2005). In the domestic situation, young horses did not show a clear spontaneous attractiveness to human tactile stimulation (Henry et al., 2006). Of course, the question arises of how the animals themselves perceive the human-defined reward: only individuals can tell what an appropriate reward is (Chance, 1992; Baragli et al., 2009). Indeed, it is not the action *per se* that is important, but the manner in which horses perceive and appraise such actions in relation to the environment and their experiences (Baragli et al., 2009). Thus, in a recent study, Sankey et al. (2010c) trained young horses to remain immobile in response to a vocal order where either food or withers' grooming were used as a reward. It appeared that only the food-rewarded horses learned the task. More intriguing was the fact that using grooming as a reward did not improve the relation between the horses and their trainer, contrarily to the use of a food reward, as revealed by the horses' behavior during a motionless person test. Therefore the use of appropriate food rewards (i.e., familiar attractive food: e.g., carrots in Sankey et al., 2010b, pellets in Sankey et al., 2011) have increased learning in all cases (McDonnell, 2000; Schultz, 2004), while there is no such report for tactile rewards. It remains to understand the processes involved in these different learning performances and relationship with the trainer.

Of course, attentional processes are likely candidates to explain these results. Since Posner's crucial experiments on attention and detection of signals, attention is defined as an ability to focus perception on one stimulus, while filtering out other simultaneous stimuli that are less relevant (Posner et al., 1980). Thus, attention, “the selective aspect of perception” (Treisman, 1969), is the mechanism, the pre-requisite for adaptive response, and a part and parcel of the process of learning (Oades and Sartory, 1997). Moreover, attentional processes are widespread amongst vertebrates and invertebrates (Giurfa, 2013). For various species, paying attention towards environmental stimuli and the salience of the stimuli are crucial to enhance learning performances (drosophila: van Swinderen and Greenspan, 2003; honeybees: Spaethe et al., 2006; non-human primates: Range and Huber, 2007; humans: Kruschke, 2003). Thus, deficit of attention (Davids et al., 2003) and also “excessive” attention may affect learning performances (Topál et al., 1997). Hence, horses may also have become so dependent upon humans that they expect humans to solve a task instead of doing it themselves: “excessive” attention towards humans led to lower learning performances (Lesimple et al., 2012). Therefore, grooming may lead to lower performances either because it is not positively reinforcing or because horses are just not paying attention to the task itself. Since feeding is the main activity of horses in natural conditions (Waring, 2003) and food a classical primary reinforcer, we expect it to elicit attention in all horses. On the contrary, since allogrooming is dependent upon horses and context, we expect its potential “hedonic” effect to vary largely between individuals and hence to enhance or decrease attention accordingly. If this is the case, we would see more variation in attention in response to grooming rewards than to food rewards and see a different relation between attention and learning between both procedures. In order to disentangle better the relation between attentional processes and learning, we focused on horses' attentional indicators (e.g., postural and behavioral adjustments: monitoring, gazing, and expressing behaviors towards the trainer) during training using either food or grooming as a reward. In particular, the duration of visual monitoring has been shown to be a useful indication to assess an animal's attention towards humans (Xitco et al., 2004; Sankey et al., 2011) or social models (Range and Huber, 2007). Interrelations between learning and attentional indicators were explored in term of rate of learning (i.e., day when each horse first reached the performance criterion of 60 s of immobility) and in terms of learning *per se* (i.e., improvement in performance: seconds of immobility on day 5 minus seconds of immobility on day 1). From a fundamental perspective, this study contributes to understand the relationship between attentional processes and cognitive performances. It may also allow the development of more efficient methods for animal training.

## Materials and methods

### Ethical statement

Data presented in this study originated from experiments performed during winter 2009 (described in Sankey et al., 2010c) and reanalysis of video recordings. Both procedure and testing were conducted in accordance with the French regulations governing the care and use of research animals. The experiment was performed in accordance with the European Communities Council Directive of 24th November 1986 (86/609/EEC).

### Animals

Study subjects were 15 Konik horses (6 females, 6 males, and 3 geldings) (Table 1), a primitive breed originating from the wild Tarpan horse (Jezierski et al., 1999). Subjects had been reared under either conventional domestic conditions (*N* = 10) or semi natural conditions in a 1600 ha forest reserve with their respective families (*N* = 5). Anyway, forest-reared foals were caught when they were about 10 months old and then kept with their stabled peers. All young horses were kept together under the same conditions, in multi-age groups, where they were able to express their natural behavioral repertoire in which grooming others' withers is considered to play a socio-positive role (Feh, 2005). Horses were 1–2 years old at the time of the experiment and housed in loose stables. No additional tactile contact with humans took place, except for daily tethering for feeding and for hoof trimming every 3 months.

**Table 1:** **Subjects (Name; Group: training group, FR, food-rewarded horses; GR, grooming-rewarded horses; Age: in years old; Sex: F, female; M, male; G, gelding; Rearing conditions until 10 months old)**.

**Subject**	**Group**	**Age**	**Sex**	**Rearing condition**
Bachar	FR	1	M	Domestic
Brzezina	FR	1	F	Domestic
Gabor	FR	1	M	Domestic
Liryk	FR	1	G	Semi-natural
Nasza	FR	1	F	Semi-natural
Niki	FR	1	M	Semi-natural
Pilar	FR	1	G	Domestic
Tropina	FR	1	F	Domestic
Prima	GR	2	F	Domestic
Jadzia	GR	1	F	Domestic
Jagna	GR	1	F	Domestic
Lipeusz	GR	1	G	Domestic
Lotnik	GR	1	M	Domestic
Nazar	GR	1	M	Semi-natural
Nonius	GR	1	M	Semi-natural

### Training procedure (see also Sankey et al., 2010c)

Sankey et al. (2010c) training procedure consisted in training the subjects to remain immobile in response to a vocal command (“Reste!”—in French—which means “stay still” in English) for an increasing duration (5, 10, 30, 45–60 s) and despite the trainer taking a step back. Training sessions, including several trials (minimum: 2 trials per day; maximum: 17 trials per day), lasted 5 min, with training performed on 5 consecutive days. Auditory signal was given just once per trial, then the trainer remained silent. The training took place in the horse's home stable (to limit stress due to social isolation), where they were tethered facing the walls and given hay *ad libitum*. For training, the experimenter led the focal horse to the center of the stable and positioned herself to its left, facing the horse. In order to distract the neighboring horses' attention (e.g., non-experimental horses kept in the neighboring stalls) from the vocal command, a white noise was broadcast via two loudspeakers placed each side of the stable, facing the tethered horses. After completion of a daily training, the horses were set free in an adjacent outdoor paddock.

Horses were randomly allocated according to sex, age, and origin (e.g., domestic or forest-reared horses) to one of two training groups:

- food-reward group (FR: *N* = 8): the experimenter hand-gave a small piece of carrot to the horse when it responded correctly to her command. The food reward was hidden from the horses during training as it was in the trainer's pocket. It was given to horses only at the end of the required immobility time.
- grooming-reward group (GR: *N* = 7): the experimenter scratched the horse's withers vigorously three times (5 s) when it responded correctly to her command.

The horse had to fulfill the performance criterion of each step to get to the next one, that is, it had to succeed three times consecutively in the given step. For example, a horse had to remain immobile on order during 5 s three times (trials) consecutively (step 1), before moving on to the next step (10 s of immobility). Horses were not limited in the number of steps they could complete successfully within a training session, which simply ended after 5 min. Each time they remained immobile for the required duration, horses of the FR group received hand-given carrots as a reward and carried on with the training program, whereas horses from the GR group had wither scratching and carried on with the next steps. For all horses (i.e., FR group and GR group), a trial was considered as failed when the horse moved before the required duration or when it showed defensive behaviors. A fail was neither punished nor a source of human attention. Thus, after failed trials, as for succeeded trials, the horse were led in hand for at least 15 s around the training arena before receiving again the vocal order and trying again. Punishment was never used in this experiment, nor was negative reinforcement. Learning performances of the two groups of horses are shown in Sankey et al. (2010c) and correspond to the maximum time step validated (i.e., three consecutive successes) each day, as well as the maximum amount of immobility time (Sankey et al., 2010c).

### Behavioral measurements

During all the training sessions, the horses' behavior was video-taped. Recordings were then analyzed using a focal sampling method: all behaviors of the focal animal were recorded continuously (Altmann, 1974) and were expressed in seconds divided by the number of trials per day. For the present study, we collected classical attentional state measurements (Xitco et al., 2004; Sankey et al., 2011): (1) time spent monitoring the trainer (monitoring was defined as the rotation of the head approximately 45° or more towards the trainer during training) vs. time spent orienting the head towards the environment (i.e., to the right, straight-forward, or downward); (2) time spent gazing at the trainer vs. gazing at the environment (a gaze was counted each time the head of the focal horse remained motionless with gaze duration longer than 1 s) (Blois-Heulin, 1999) and binocular gazes were associated with forward ears (Brajon et al., in preparation); and (3) all behaviors directed towards the trainer (i.e., licking, nostril sniffing, nibbling, chewing) vs. “agitated” behavior (i.e., moving forward or backward).

### Relation between learning and attention

Interrelation between learning performances found in Sankey et al. (2010c) and attentional indicators were explored in two ways. (1) The relationship between rate of learning: rapidity to first reach the performance criterion (i.e., day when each horse reached 60 s of immobility) and the overall trainer-directed attentional state of each horse (i.e., score of mean percentage of time of gazing, monitoring, and expressing behaviors towards the trainer during all training days). (2) The relationship between learning *per se* (i.e., improvement in performance: seconds of immobility on day 5 minus seconds of immobility on day 1) on the first day and the last day of training and visual trainer-directed attention (i.e., gazes towards the trainer/number of trial on these days).

### Statistical analysis

As data were not normally distributed, we used non-parametric statistical tests (Siegel and Castellan, 1988). Fisher's exact tests were used to compare sex, age, or rearing conditions composition between groups after being randomly assigned and no difference appeared (Fisher's exact test: *P* > 0.05 for all). Friedman test and Wilcoxon signed rank *t*-tests were used to compare matched paired data (i.e., comparison of the behavior of the same individual at different times, for example between the beginning and the end of training). Multiple pairwise comparisons using Wilcoxon signed rank test had a *p*-value adjustment with fdr (“false discovery rate”) method. Mann-Whitney U-tests were used to compare the two experimental groups in terms of number and duration of trials per day and no difference appeared (Mann–Whitney U-test, *p* > 0.05 for all days) and in terms of monitoring, gazing and expressing behavior towards the trainer. The relationship between attentional state and the rate of learning was evaluated by plotting the attentional state of each horse (i.e., score of mean percentage of time of gazing, monitoring, and expressing behaviors towards the trainer during all training days) as a function of the day when it first reached the performance criterion (i.e., immobility during 60 s). A two-sample Kolmogorov–Smirnov test (KS) was used to compare distribution between food-rewarded horses and grooming-rewarded horses. The relationship between visual trainer-directed attentional state (i.e., gazes towards the trainer) and learning *per se* (i.e., improvement in performance: seconds of immobility on day 5 minus seconds of immobility on day 1) on the first day and the last day of training was evaluated by Spearman's correlation test. These analyses were run with Statistica 7.1 software ©and R software (accepted *p* level at 0.05 R Development Core Team, 2011; Maxime, 2013).

## Results

### Overall attentional state

**Figure 1:**
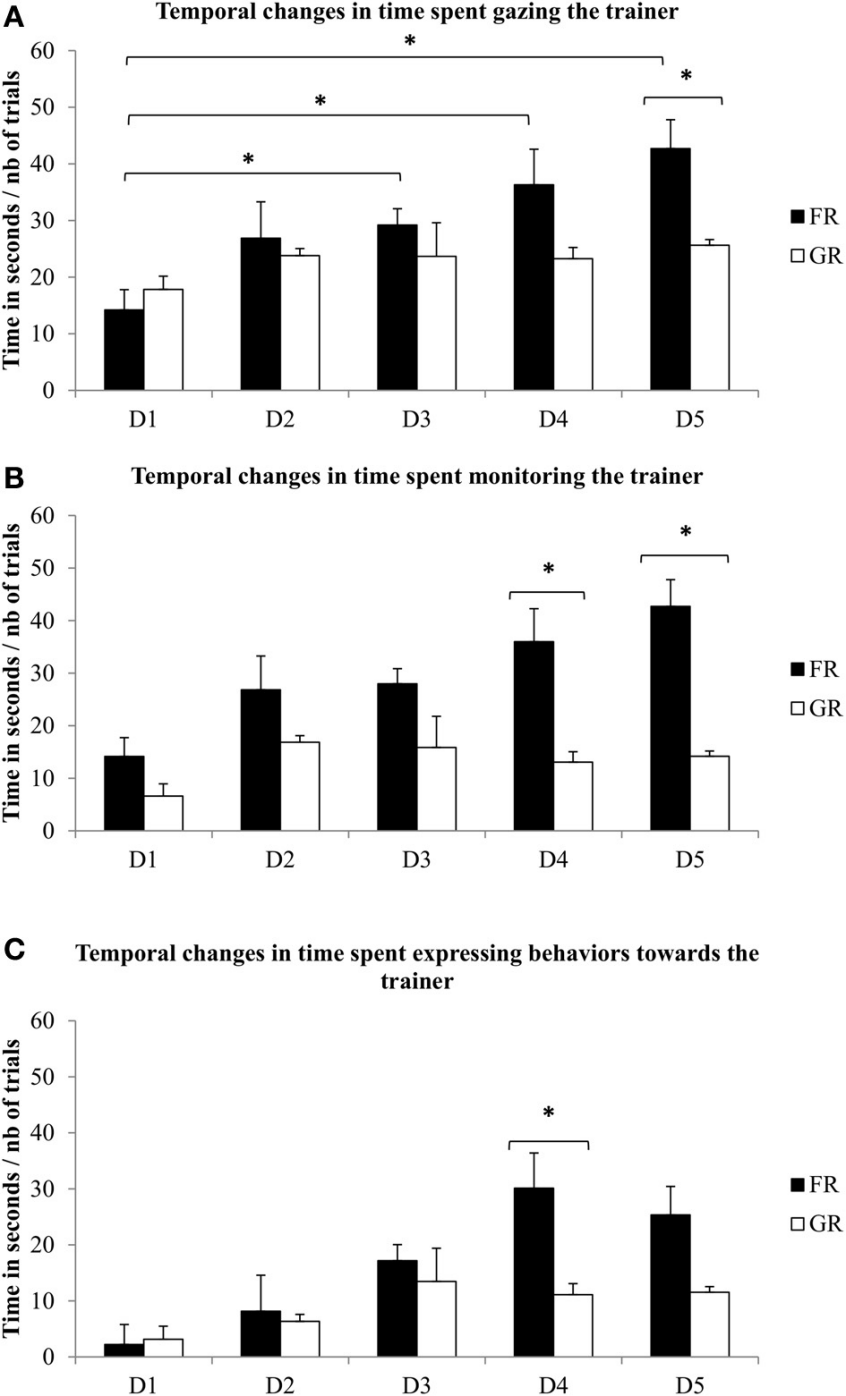
**Attentive behaviors**. Time spent **(A)** gazing, **(B)** monitoring an **(C)** expressing behaviors towards the trainer by Food-reinforced (FR: *N* = 8) (Friedman test and Wilcoxon test *P* < 0.05) and by Grooming-reinforced (GR: *N* = 7) horses (Friedman test *P* > 0.05) during the 5 training days (in seconds/number of trial per day). Differences between both group increases on day 4 and 5 for all parameters, going towards more intragroup homogeneity and differences between groups on these days (Mann-Whitney U-test *p* < 0.05). Error bars represent standard errors. ^*^P < 0.05.

Clear differences in horses' attentional behaviors towards the trainer could be observed in relation to type of reward and during the training course. Whereas no differences between the two experimental groups could be evidenced on the first day of training, durations of gazes directed towards the trainer changed during the training course for the Food-rewarded (FR) horses (Friedman test _(*N* = 8, *df* = 4)_ = 20, *P* = 0.0005) (Figure 1A), with lower attention in the first day compared to the third, fourth, and last day of training (Wilcoxon signed-rank test with fdr correction: *X*_D1_ ± *SE* = 14.2 ± 3.6 s, *X*_D3_ ± *SE* = 29.2 ± 2.9 s, *n* = 8, *P* = 0.03; *X*_D4_ ± *SE* = 36.3 ± 6.3 s, *n* = 8, *P* = 0.03; *X*_D5_ ± *SE* = 42.7 ± 13.4 s, *n* = 8, *P* = 0.03) and with higher attention in the last day compared to the second and third day of training (Wilcoxon signed-rank test with fdr correction: *X*_D5_ ± *SE* = 42.7 ± 13.4 s, *X*_D2_ ± *SE* = 26.9 ± 6.4 s, *n* = 8, *P* = 0.03; *X*_D3_ ± *SE* = 29.2 ± 2.9 s, *n* = 8, *P* = 0.03). No differences between days were found for the Grooming-rewarded (GR) horses (Friedman test _(*N* = 7, *df* = 4)_ = 4.2 *P* = 0.37) (Figure 1A). Similarly, FR horses' monitoring of their trainer and their behavior towards the trainer changed during the training course (monitoring the trainer: Friedman test _(*N* = 8, *df* = 4)_ = 13.8, *P* = 0.008; behaviors towards the trainer: Friedman test _(*N* = 8, *df* = 4)_ = 15.8, *P* = 0.003), whereas that of GR horses did not (monitoring the trainer: Friedman test _(*N* = 7, *df* = 4)_ = 4, *P* = 0.41; behaviors towards the trainer: Friedman test _(*N* = 7, *df* = 4)_ = 7.2, *P* = 0.12) (Figures 1B,C).

**Figure 2:**
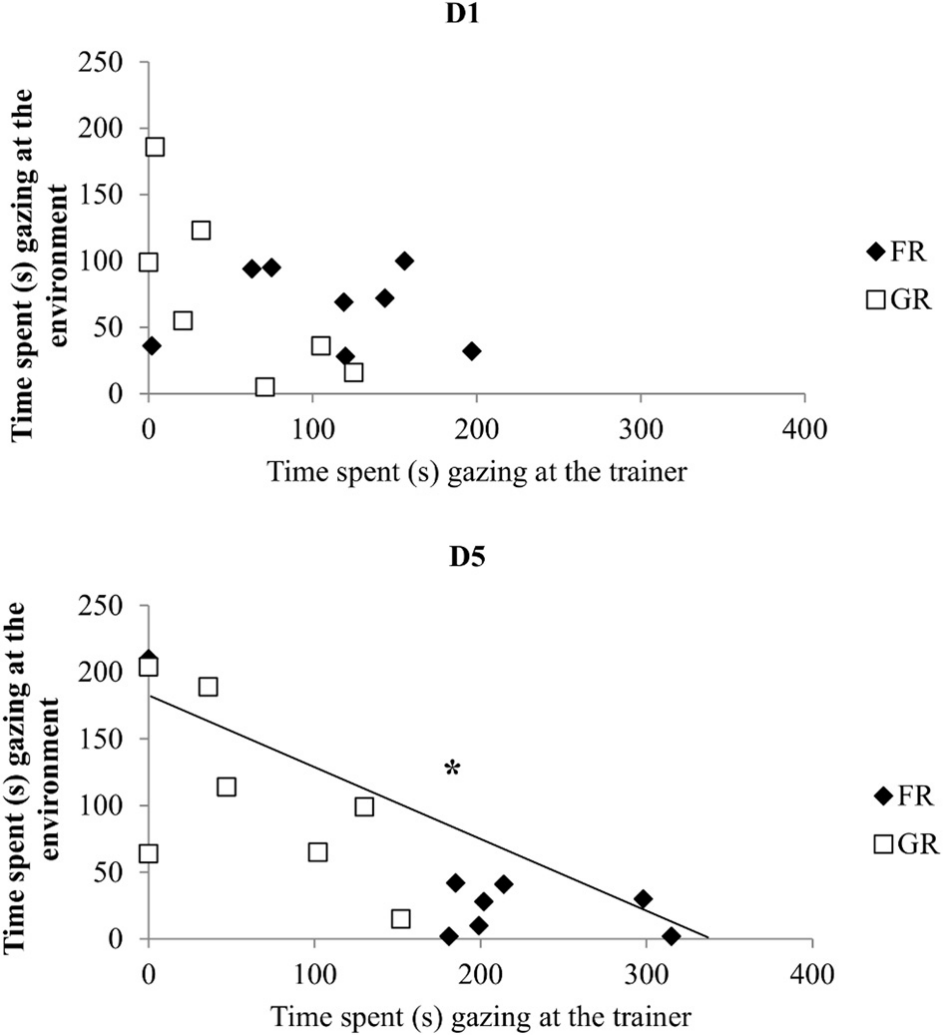
**Interrelation between observing the trainer and observing the environment**. Correlation between times (in seconds) spent gazing at the trainer vs. gazing at the environment along training (Spearman's correlation test, ^*^p < 0.05). FR, food rewarded horses; GR, grooming-rewarded horses. D1 represent the first day of training, D5 represent the last day of training.

Differences between both groups increased on day 4 for all parameters, reaching clear statistical significance on day 4 and/or 5 which reflects an increased intragroup homogeneity. (Figures 1A–C). Thus, on day 4 and/or 5, FR horses directed more behaviors towards the trainer than did GR horses: they spent more time gazing (Mann–Whitney U-test on day 5: *X*_FR_ ± *SE* = 42.7 ± 5.1 s, *X*_GR_ ± *SE* = 25.6 ± 1 s, *U* = 2, *P* = 0.02; Figure 1A), monitoring the trainer (Mann–Whitney U-test on day 4: *X*_FR_ ± *SE* = 35.9 ± 6.3 s, *X*_GR_ ± *SE* = 13.1 ± 7.8 s; *U* = 1, *P* = 0.01; Figure 1B Mann–Whitney U-test on day 5: *X*_FR_ ± *SE* = 43.7 ± 5 s, *X*_GR_ ± *SE* = 14.2 ± 4 s; *U* = 1, *P* = 0.01; Figure 1B), and expressing behavior towards the trainer (Mann–Whitney U-test on day 4: *X*_FR_ ± *SE* = 30.1 ± 6.9 s, *X*_GR_ ± *SE* = 11.1 ± 4 s; *U* = 2, *P* = 0.02; Figure 1C). Conversely, GR horses spent the same amount of time gazing at (Friedman test _(*N* = 7, *df* = 4)_ = 2.8, *P* = 0.59) and monitoring the environment (Friedman test _(*N* = 7, *df* = 4)_ = 4, *P* = 0.41) over the whole set of sessions while FR horses showed a decrease until the third day (gazing: Friedman test _(*N* = 8, *df* = 4)_ = 10.2, *P* = 0.03; monitoring: Friedman test _(*N* = 8, *df* = 4)_ = 12.6, *P* = 0.01) and showed less time gazing (day 4: 10% of time; day 5: 21% of time) and monitoring (day 4: 10% of time; day 5: 21% of time) the environment on day 4 and 5 compared to gazing (day 4: 90% of time; day 5: 79% of time) and monitoring (day 4: 90% of time; day 5: 79% of time) the trainer. Thus, significant negative correlation between gazing at the trainer and gazing at the environment could be found in the second, fourth and last day of training (Spearman's correlation test: D1, *N* = 15, *rs* = −0.33, *p* > 0.05; D2, *rs* = −0.82, *p* < 0.05; D3: *rs* = −0.51, *p* > 0.05; D4: *rs* = −0.55, *p* < 0.05; D5: *rs* = −0.78, *p* < 0.05, Figure 2). For FR horses and GR horses distinctively, significant negative correlation between gazing at the trainer and gazing at the environment could be found in the second day (Spearman's correlation test: D2, *N*_FR_ = 8, *rs* = −0.76, *p* < 0.05; *N*_GR_ = 7, *rs* = −0.86, *p* < 0.05). Thus, on day 3, both groups differed significantly with FR horses being less attentive to the environment (gazing: Mann–Whitney U-test: *X*_FR_ ± *SE* = 1.9 ± 0.7 s, *X*_GR_ ± *SE* = 7.8 ± 2.3 s; *U* = 3, *P* = 0.04; monitoring: Mann–Whitney U-test: *X*_FR_ ± *SE* = 1.8 ± 0.7 s, *X*_GR_ ± *SE* = 7.8 ± 2.3 s; *U* = 3, *P* = 0.04). Although not statistically significant, FR horses showed a decrease of agitated behaviors (Wilcoxon signed-rank test: *X*_D1_ ± *SE* = 33.7 ± 9.2 s, _D5_ ± *SE* = 8.1 ± 4.2 s, *n* = 8, *P* = 0.09) while no clear change over time could be observed in GR horses who showed a similar or higher time spent expressing agitated behaviors at D5 (Wilcoxon signed-rank test: *X*_D1_ ± *SE* = 11.8 ± 4.3 s, *X*_D5_ ± *SE* = 15.6 ± 6.5 s, *n* = 7, *P* = 0.83).

### Relation between attention and learning

**Figure 3:**
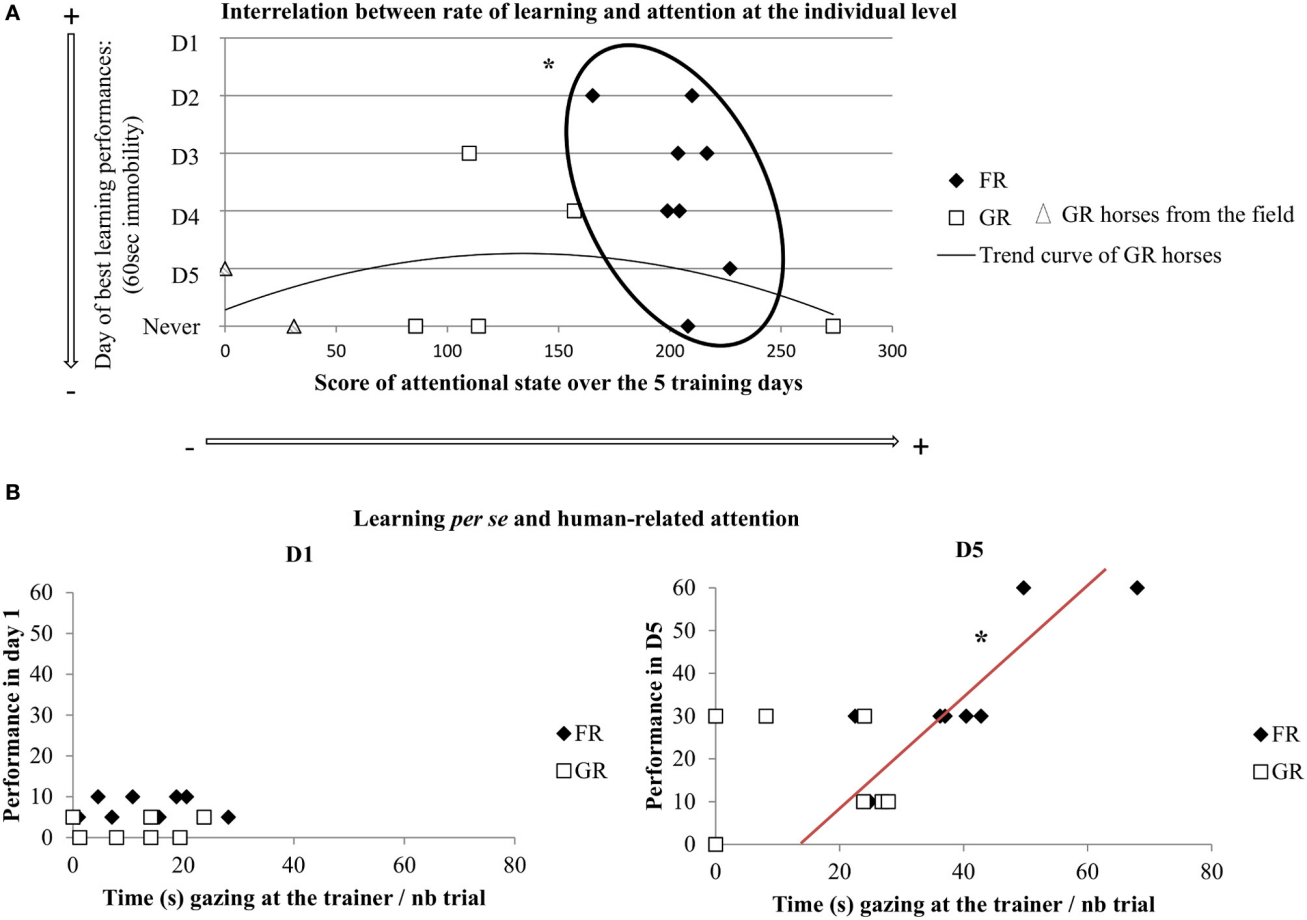
**Interrelation between learning and attention. (A)** Individual plot of the rate of learning: rapidity to first reach the performance criterion (i.e., day when each horse reached 60 s of immobility) and the overall trainer-directed attentional state of each horse (i.e., score of mean percentage of time of gazing, monitoring, and expressing behaviors towards the trainer during all training days); FR, food rewarded horses; GR, grooming-rewarded horses. FR horses showed a “window” of attention related to faster learning performances, GR horses' pattern follow an almost normal curve where the extreme animals (i.e., highest and lowest attention) had the slowest learning performances (KS test, ^*^p < 0.05). **(B)** Correlation between learning *per se* (i.e., step validated by each horse in day 1: 5, 10, 30, 60 s of immobility) and visual trainer-directed attention (i.e., gazes towards the trainer/number of trial on these days) (Spearman's correlation test, ^*^p < 0.05). FR, food rewarded horses; GR, grooming-rewarded horses. D1 represent the first day of training, D5 represent the last day of training.

**Figure 4:**
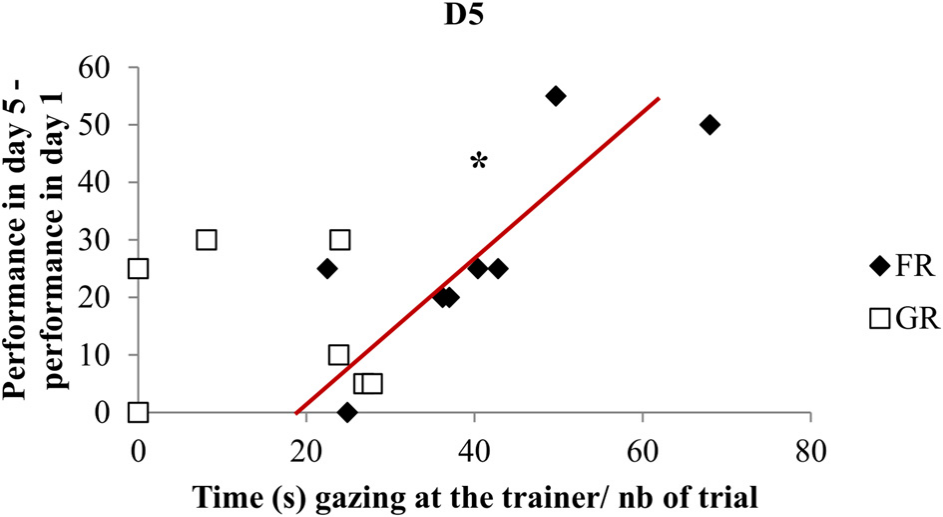
**Interrelation between improvement in learning performances and attention**. Correlation between improvement in learning performance in day 5: seconds of immobility on day 5 minus seconds of immobility on day 1 and visual trainer-directed attention (i.e., gazes towards the trainer/number of trial on these days) (Spearman's correlation test, ^*^p < 0.05). FR, food rewarded horses; GR, grooming-rewarded horses. D5 represent the last day of training.

Firstly, plotting the rate of learning (i.e., the day when each horse first reached the performance criterion: 60 s of immobility) and attentional measures (i.e., mean percentage of time gazing, monitoring, and expressing behaviors towards the trainer over all training days) revealed clear differential distribution between both groups of horses (K-S test, *p* < 0.01) (Figure 3A). Thus, all food rewarded horses were lumped in a tight relation with high level of attention towards the trainer in relation to faster learning. On the contrary, the curve showed high variability of grooming-rewarded horses in terms of attention, with some horses showing extreme slow learning. Interestingly, the two GR horses originated from the field (“semi-natural horses”) are those that paid least attention towards the trainer, while the three FR “semi-natural horses” were just lumped with the domestic-raised FR horses. Secondly, plotting the relationship between visual trainer-directed attentional state at the beginning (i.e., day 1) and the end (i.e., day 5) of training with learning *per se*, revealed that there was no correlation on the first day of training. This situation on day 1 reflects a lack of relation between obeying (not much at that stage) and attention towards the trainer and was very different from that observed on day 5. After horses had had 5 sessions of training, a clear correlation appeared for FR horses showing that the more attentive horses had also better learning (Spearman's correlation test: *N* = 8, *rs* = 0.78, *p* < 0.05), (Figure 3B). Interrelation between improvement in learning performance in day 5 (i.e., seconds of immobility on day 5 minus seconds of immobility on day 1) and visual trainer-directed attention (i.e., gazes towards the trainer/number of trial on these days) revealed that a clear correlation appeared for FR horses showing that the more attentive horses had also better improvement in learning performances (Spearman's correlation test: *N* = 8, *rs* = 0.72, *p* < 0.05), (Figure 4).

## Discussion

The comparison of task related attention according to the type of reward used in positive reinforcement training revealed clear overall differences: along training days, horses showed a real increase of attention towards the trainer in the case of a food reward while no such enhancement of attention was observed for a tactile reward at the group level. These results confirm those obtained in terms of learning performance on these same horses by Sankey et al. (2010c): FR horses were the only ones to showed an increase of performance over sessions. Grooming reward was associated with more inattentive visual responses (i.e., head orientation towards the environment) and no decrease of “agitated” behavior (i.e., moving forward or backward). Relationship between learning and attentional state revealed that fast learners are within an “optimal window of attention” promoted by food reward whereas slow learners showed individual variation of attention (i.e., too little or too much attention). Moreover, learning was influenced by attention which was highly visible at the end of the training where the more attentive horses (which were FR horses) had also better learning performances. Since the present study was based on an earlier dataset it was not possible to gather data on longer training periods. The increasing pattern of differences between both groups until clear differences on days 4 and/or 5 suggests that it would be worth in further studies to study longer time periods. Moreover, the trends of differences appeared clearly from day 3 and 4 on which may be a crucial step in training. In another training study, Sankey et al. (2010b) had shown that horses have anticipations on the quality of training sessions from day 3 on. Actually, the main difference between both procedures was the homogeneity of responses of FR horses as compared to GR horses. This was clearly reflected by the curves relating human-directed attention and rate of learning. Most GR horses showed lower attention and performances than FR horses, or on the contrary showed extremely high attention towards the trainer but slow performances, reminding of Lesimple et al. (2012)'s findings where horses had their attention focused on humans' as they relied upon them to solve a cognitive task. In the same way, dogs that were more dependent to their owners (more glancing and following) showed also decreased problem solving abilities in a cognitive task (Topál et al., 1997). Interestingly, in our study this “excessive” attention concerned a domestic raised horse that was hence used to human provisioning and to human positive actions since an early age. “Semi-naturally” raised horses showed much less interest for humans in the GR group, which may be related to the fact that their only other direct contact with human had been catching from the field followed by separation from their dams and peers. Another two GR horses showed both intermediate levels of attention and good learning performances. Therefore, it seems that there could be an “optimal” window of human-directed attention that favors learning of the task, while lowered or “excessive” attention prevents making the right association between the human and the task to be learned. On the other hand, learning was influenced by attention which was highly visible on day 5. At the beginning of training, there was a lack of relation between obeying and attention towards trainer but at the end of training, the more attentive horses (which were FR horses) had also better learning performances.

This study, showing that grooming reward did not enhance attentional state in horses, is in line with earlier findings that a grooming reward cannot be considered as a primary reinforcement for horses as it did not enhance the learning performances either (Sankey et al., 2010c). Grooming the withers therefore does not appear to be an efficient reward for horses. One first explanation could be that here grooming by a human handler was performed during 5 s which could be too short to elicit an heart rate decrease, contrary to 3-min grooming that has been shown to decrease horses' heart rate (Feh and De Mazières, 1993). Nevertheless, it has been previously shown that even 10 min of gentle tactile stimulation is not forcefully perceived as a positive event by foals (Henry et al., 2006, 2009). One has also to consider that a decrease in heart rate does not mean that it is perceived sufficiently positively to be considered as reinforcement and thus promote learning (Sankey et al., 2010c). Another explanation could be that the rewarding effect of grooming may not be immediate enough for establishing the link between the horse's required behavior and the reward: unlike food reward which had immediate benefits, grooming reward could be coupled with a temporal contiguity problem (Schultz, 2004). The main hypothesis is that in horses, physical contact is less crucial in terms of survival than food as it is very restricted through occasional licking of the young by its dam and adult mutual grooming, which only represents 2–3% of their time-budget and is more frequent at some times of year (e.g., moulting) (Boyd et al., 1988). Although grooming the withers is considered to play a positive social role, grooming by a human may depend upon time of year and/or may not create a sufficiently positive emotion to have a rewarding value and to promote attention and learning. The grooming rewarded horses' behavior expressed in the present study are similar to those of non-reinforced horses observed in other studies using the same procedure both in terms of performance (Sankey et al., 2010a) and attention (Brajon et al., in preparation). Of course, tactile stimulation may become positive if associated with a primary reward such as food, becoming a secondary reinforcement. In contrast, all food rewarded horses showed an increased performance (Sankey et al., 2010a,b,c) and attention (i.e., more gazes, ears, neck, and sniffing towards the trainer) (Brajon et al., in preparation) in different studies using different breeds (i.e., French saddlebreds, Koniks) and/or types of food (i.e., pellets, carrots). Our study confirms that horses are able to learn human words and the corresponding expected task. These findings therefore seem to rely upon general principles that relate the positive, neutral, or negative valence of stimuli associated with the learning task. Thus the emotional content of an event can modify and update the goals and consequently alter the direction of attention to a stimulus (Taylor and Fragopanagos, 2005). On the contrary, excessive motivation may lead to inappropriate behaviors that prevent learning or inhibit flexibility (Quay, 1997). This emotional, rewarding or punishing, content may modulate visual selection and therefore selective attention (Raymond, 2009). On one hand, stimuli carrying a positive emotional charge may increase the representational strength of these stimuli and thus enhance motivation to perform the requested response (Taylor and Fragopanagos, 2005). For instance, motivating stimuli (e.g., monetary reward) attract and hold humans' attention in learning tasks better than neutral stimuli (Krapp, 1999; Libera and Chelazzi, 2006). This may also be the case when food reinforcement is used to train horses. Indeed, in animal behavioral studies, motivation is defined as “a construct used to describe the strength or willingness with which an animal engages in behavior” (Toates, 1986, cited by Kirkden and Pajor, 2006). Thus, earlier findings showed that the few short food mediated interactions promote learning and memory but also a positive relationship with humans both at short and long term: horses trained with a food reward approached sooner and remained closer to humans during human-animal relation tests after and outside training (Hausberger et al., 2008; Sankey et al., 2010a,b,c), therefore suggesting that positive emotions and motivation are involved. On the other hand, emotional stimuli carrying a negative charge induce various responses. Indeed, studies using aversive conditioning provided contrasting results. Stormark and Hugdahl (1997), training human subjects to perform a spatial orienting task (Posner et al., 1982), found that the subjects' attention moved away from the location of a cue faster when that cue was aversive. In contrast, Armony and Dolan (2002) using a similar task found that frightening cues captured subjects' attention, and this led to difficulties in shifting attention to the correct location. Sankey et al. (2010b) found that horses negatively reinforced during a learning task had an increase of heart rate, made more head movements and less gazes towards their trainer whereas horses positively reinforced had no heart rate increase and low and round neck position, suggesting a calmness state and a positive perception of the situation (von Borstel et al., 2009). In our study, punishment was never used, nor was negative reinforcement, and hence could not impact horses' attention.

Finally, the finding that food rewards have durable beneficial effects on most horses' learning performances and relationships with humans in Sankey et al. (2010c) may be linked to the motivational and hence attentional processes generated by the trainer who gives the food reward and therefore promotes learning of the association between her and the positive valence of the situation. In other words, since horses' selective attentional state increased in the presence of a food reward, they focused on the important input (e.g., the trainer and her orders) and thus showed better learning performances and durable positive relationships with humans as revealed by Sankey et al. (2010c), linked with positive emotions and motivation due to the positive valence of the situation. Moreover, neuronal substrates such as lateral prefrontal (for detecting changes in the external environment, Knight et al., 1995), superior and inferior parietal cortex (for spatial representation and updating, coordinate transformation, as well as abstract motor planning, Behrmann et al., 2004), and anterior cingulate gyrus (for motivational aspect, Nebel et al., 2005) are involved in tasks of focused attention in humans (Nebel et al., 2005). These structures are supposed to form the basis of a higher attention related network which is linked with encoding relevant and motivated information for an individual and then enhancing their learning and memorization performances (Treisman, 1969). Visual indicators of attention were used even if the trainer gave an auditory signal. This is in accordance with earlier studies showing that auditory inputs may, according to their significance, elicit visual exploration (Basile et al., 2009; Lemasson et al., 2009; Proops et al., 2009). Actually, visual attentional behavior such as gazes (and especially mutual gazes) can also be associated with trying to identify a human's intentions or expectations (Sankey et al., 2011; Lesimple et al., 2012) or may be part of the development of the building of a positive relationship, as observed in some primate species (Micheletta and Waller, 2012). It may also reflect as well the emotional and mental state of the subject (Emery, 2000).

This study, based on horses, reveals the complex pattern relating attentional processes to learning performances. It shows that whereas primary rewards do increase both attention and performances in most individuals, other rewards may have effects varying according to the individual's sensitivity and life experience. Most of all it shows that there is a window of “optimal attention” that promotes learning per session and for all training, whereas too little or “excessive” attention leads to worse learning performances. Thus, learning performances appears to be mediated by an optimal attention promoted by food reward. These results open new lines of thought about individual sensitivities (only individuals can tell what an appropriate reward is), attentional processes and learning.

### Conflict of interest statement

The authors declare that the research was conducted in the absence of any commercial or financial relationships that could be construed as a potential conflict of interest.
